# Peripheral blood persistence and expansion of transferred non-genetically modified Natural Killer cells might not be necessary for clinical activity

**DOI:** 10.1093/immadv/ltac024

**Published:** 2023-01-13

**Authors:** Lucia Silla

**Affiliations:** Universidade Federal do Rio Grande do Sul – Hospital de Clinicas de Porto Alegre, Instituto Nacional de Ciência e Tecnologia REGENERA, Porto Alegre, Brazil

**Keywords:** NK cell, lymphocyte adoptive immunotherapy, acute myeloid leukemia, clinical trial

## Abstract

Natural killer (NK) cells are innate lymphocytes that react without previous exposition to virus infected or malignant cells and stimulate adaptive immune response to build a long-lasting immunity against it. To that end, tissue resident NK cells are predominantly regulatory as opposed to cytotoxic. In the hematopoietic stem cell transplant (HSCT) setting, which curative potential relies on the graft versus leukemia effect, NK cells are known to play a significant role. This knowledge has paved the way to the active investigation on its anti-tumor effect outside the stem cell transplant scenario. Based on the relevant literature on the adoptive transfer of non-genetically modified NK cells for the treatment of relapsed/refractory acute leukemia and on our own experience, we discuss the role of donor cell peripheral blood persistence and expansion and its lack of correlation with anti-leukemia activity.

## Graphical Abstract

**Graphical Abstract:**
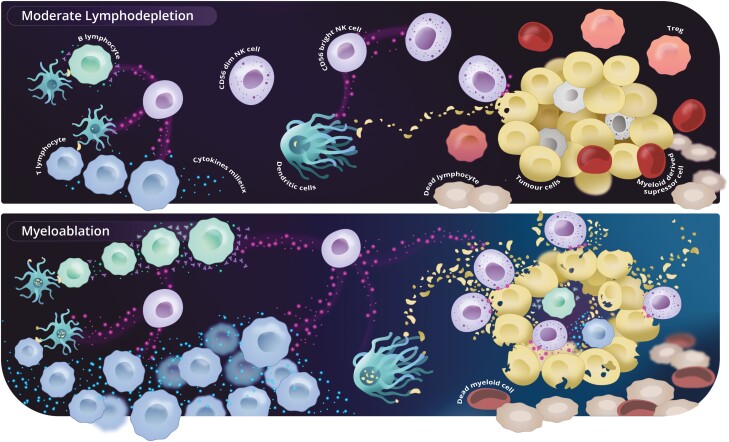

## Introduction

### Natural killer cells

NK cells are the best characterized innate lymphocyte representing 5–20% of peripheral blood lymphocytes. As innate effectors against virus-infected, cancer and stressed cells, NK cells limit viremia and tumor burden before the onset of adaptive T and B cells response. This NK cell burst subsides at days 5–7 when the adaptive immune response leads to microorganism clearance and probably to tumor eradication in a successful immune reaction [1].

Although NK cells are typically recognized as cytotoxic only, their regulatory function has increasingly been accepted and should be carefully examined. There are two major NK subsets: CD56^bright^ and CD56^dim^ – immature and mature NK cells, respectively [2]. This terminology, however, refers to ontogeny, not to the ability to effectively react against disturbed cells since both subsets can do it in different ways. CD56^bright^ reacts by secreting various cytokines, chemokines, and growth factors while CD56^dim^ is cytotoxic and one of the major IFN-γ producing cells [3]. Both subtypes react without previous exposure, utilizing different sets or different densities of the same receptors to destroy target cells and secrete mediators (or both) related to innate and adaptive immune cells’ ­development and activation [4]. In animal models, there are different reactive functions and/or phenotype compositions between these two major subsets that assure a high variability and adequate immunosurveillance [2].

NK cells are constitutively in a pre-activation state, and as such can react against a target cell in a matter of hours. This natural and quick activation is modulated by the presence of inhibitory and stimulatory receptors such as the *Killer Immunoglobulin like Receptors* (KIR). In addition to other ligands, inhibitory KIRs for self-HLA class I ligands protect normal cells from being destroyed – the missing-self theory [5], provided that these cells are not abnormal enough to express higher levels of activating ligands or downregulate its HLA Class I expression. The set of KIRs of an individual, known as KIR haplotype, can exhibit a lower or a higher number of KIR stimulatory receptors – A and B Haplotype, respectively. Understanding the net effect of a specific KIR haplotype in combination with the minor HLA molecules receptors in addition to the natural occurring receptors (also inhibitory or stimulatory) that NK cells also express is challenging. Furthermore, other factors also may play a role in the modulation of this interactions, including the nature of the microenvironment milieux, target cells [6], nearby cells as regulatory T cells (Tregs), and myeloid derived suppressor cells [7]. In simplified systems, as *in vitro* studies or immunodeficient animal models, the main inhibitory or stimulatory set of receptors have been studied according, respectively, to the absence or presence of its related ligand on the target cell – the so-called KIR mismatch (KIR MM); upon information derived from those studies, models of ideal NK cell/target combination are being tested to predict NK cells alloreactivity.

### Tissue resident NK cells

CD56^bright^ NK cells display tissue homing markers, such as CCR7 and CD62L, [8] and are the prominent tissue resident NK cells (trNK) [4]. CD56^bright^ NK cells are found in the lungs, for example, and have been shown to react to Influenza 1 virus-infected cells, activating adaptive immune responses such as CD8 T cytotoxic and CD4 Th1 cells through IFN-γ secretion [9]. In the human liver, they account for 50% of the total lymphocytes. Among other functions, they interact with hepatocyte HLA-E via NKG2A inhibitory receptors, releasing TGF-β that prime dendritic cells (DC) to expand T CD4 regulatory cells [10]. In lymph nodes, CD56^bright^ NK cells are known to interact with DC releasing IFN-γ to drive CD4 Th1 differentiation [11]. Approximately 25% of total lymphocytes in the human kidney are CD56^bright^ NK cells. Renal trNK cells are involved in various kidney diseases [12], and in the uterus, uNK cells – known as *super-bright* NK cells – represent more than 70% of uterine lymphocytes and are the subject of several studies linking it to trophoblast remodeling. In contrast, the emergence of CD56^dim^ NK cells is linked to spontaneous abortion [13].

CD56^bright^ NK cells decline with age and it has been suggested that this decline impairs the ability to mount an adaptive T and B cell response to new antigens [14]. Older age is a risk factor for severe or lethal COVID-19 diseases. NK cell functional impairment and a decline in circulating CD56^bright^ NK cells has been shown to be associated with COVID-19 disease severity and death [15]. These findings could indicate a key role for NK cells, particularly CD56^bright^ NK cells, in stimulating adaptive immune response.

### NK zcell anti-tumor activity

Anti-malignancy NK cell activity has been documented in several *in vitro* studies. Cultured in certain conditions, NK cells can kill virtually every tumor cell line tested [16].

NK-cell adoptive immunotherapy has been studied in myeloid malignancies based on clinical data in Hematopoietic Stem Cell Transplantation (HSCT). The Graft versus Leukemia effect (GVL) is recognized as the cellular mechanism through which HSCT can cure leukemia [17]. However, GVL is also associated with the presence of graft-versus-host disease (GVHD) and its related morbidity and mortality. In a study on T depleted haploidentical HSCT (HHSCT) for the treatment of high-risk acute myeloid leukemia (AML), the GVL effect mediated by KIR-MM haploidentical NK cells was shown to occur in the absence of GVHD [18]. It is now known that NK cells play a significant role in allogeneic HSCT’s GVL effect [19] that is reinforced by the demonstration that NK cells’ early recovery following HSCT is ­associated with fewer relapses and improved survival [20]. HSCT-related GVL effect can cure hematological malignancies and to obtain it without GVHD would be ideal.

### NK cells alloreactivity in HSCT

Although in the T depleted haploidentical HSCT scenario additional studies have confirmed the benefits of utilizing KIR-MM donors, the benefit of donor recipient NK alloreactivity in predicting outcomes in T replete haploidentical HSCT or in the HLA match unrelated T repleted HSCT setting is less clear, with some studies suggesting NK alloreactivity is associated with better outcomes and some suggesting it is not [21–24]. The increased complexity of these HSCT strategies could probably explain such conflicting findings.

### NK cell adoptive immunotherapy

Several investigators are studying KIR MM haploidentical NK cell immunotherapy to separate GVL from SCT and its associated morbidity. In this setting, in addition to NK cell characteristics, there are many unsettled issues such as ideal number of cells infused, preparative regimens, and so on that are being tested in phase I/II studies most of which include patients with relapsed or refractory diseases.

As can be seen in Table 1, NK cells adoptive immunotherapy feasibility, safety, and clinical efficacy has been investigated outside the HSCT setting, utilizing different NK cell products, lymphodepletion, and interleukin (IL) regimens. Interleukin (IL) is usually administered during the 14 days following cells infusion, aiming to activate and expand the donor NK cells *in vivo*.

**Table 1.** Clinical trials of non-genetically modified NK cell adoptive immunotherapy

Author	# Patients	Lymphodepletion	Product activation	NK cells/infusion	# infusion/*in vivo* activation	ORR %	CR %	NK PB peak day	% Donorcells Chimerism	Day of Chimerism detection	NK expansion % Patients	HSCT %/(#patients)	Disease	NOTES
Miller (25)	19	Cy/Flu 2×/5×	Overnight IL2	2 × 10^7^	1/IL-2	26	26	7	1 to 8	D5 D138*	NA	no	R/R AML	
Curti (28)	13	Cy/Flu 1×/5×	none	2.7 × 10^6^	1/IL-2	46	23	10	2 to 18	D28	NA	no		R/R AML (5) CR MRD+ (2) CR (6)
Bachanova (26)	42	Cy/Flu 2×/5×	Overnight IL2	3.4–3.6 × 10^6^	1/IL-2	21	12	7–14	10	D7(?)	10	9.5% (4)	R/R AML	
	15	Cy/Flu 2×/5×	Overnight IL2	2.6 × 10^7^	1/IL-2	53	20	7–14	66 D7 D14**	D7 D14	33	40% (6)	R/R AML	IL-2 DT
Dolstra (34)	11	Cy/Flu 4×/4×	cultured CB NK (IL2, IL15)	3.0 × 10^76^	1/no	***	***	8	21 PB D14 3.5 BM D7	D7 D14	NA	no	CR HR AML	
Bj€orklund (35)	16	Cy/Flu 2×/4× TBI	Overnight IL2	6.7 × 10^6^	1/no	37.5	25	7–14	41		NA	31% (5)	R/R AML	
Cooley (27)	26	Cy/Flu 2×/5×	Overnight IL15	3.4–9.6 × 10^6^	1/IL-15 IV	32	24	7–14	100 D7 42 D14	D7 D14	36	26.5% (11)	R/R AML	
	16	Cy/Flu 2×/5×	Overnight IL 15	2.6 × 10^7^	1/IL-15 SC	40	6	7–14	100 D7 33 D14	D7 D14	27		R/R AML	56% CRS
Romee (37)	9	Cy/Flu 2×/5×	Memory NK cell****	0.5—10 × 10^6^	1/IL-2	55	45	7–14	90	D7	NA	no	R/R AML	****Culture IL15, IL12.IL18
Vela (31)	20	HD CT	mboIL-15 K562	6.76 × 10^6^	1 & 2/Il-2	72	72	7	1-3****	NA	NA	50% (10)	R/R AML+	T-ALL B-ALL BP - AL
Silla (32)	13	HD CT	mboIL-21 K562	9 × 10^6^	6/no	78.6	50	7	NA	NA	NA	38% (5)	R/R AML	

*1 patient detected up to 138 days.

** D7 median 66% (0-99) D14 33% of the patients had donor NK cells inferred from the fact that to calculate donor cell expansion at day 14, chimerism % was necessary but it was not provided separately).

*** DFS 22 months (12–60).

**** In 1 patient it was 95% of donor cells.

Cy: cyclophosphamide; Flu: Fludarabine; TBI: total body irradiation; Vela HD CT: GLOVE/Ida-FLAG/Cy/Flu; Silla HD CT: FLAG; CB: Cord Blood; mboIl-15: membrane bond IL-15 K562 cells; mboIl-21: membrane bond IL-21 K562 cells; IL-2: Interleukin 2; IL-15: Interleukin 15; IL-21: Interleukin 21; DT-IL-2: Diphtheria Toxin bond IL-2 for Treg in vivo depletion; BP-AL: biphenotype acute leukemia. NA: not available.

The clinical activity of the adoptive transfer of moderately activated haploidentical NK cells (IL-2 overnight) in a population of relapsed/refractory AML (R/RAML) patients led to 26% of ORR and CR [25]. In this trial, which also included patients with solid malignances, Miller et al. tested low and moderate intensity lymphodepletion strategies, followed by IL-2, for which off-target effects were mild and manageable. Immune reconstitution studies showed that in the patients who received moderate intensity of Cy/Flu, but not in those receiving low-dose lymphodepletion, donor NK cells peaked around day +7 followed by a progressive decrease. This was a very important study because in addition to the observed anti-leukemia activity, it suggested that lymphodepletion intensity can influence adoptive NK cell immunotherapy clinical activity and was the first to show transient NK cell ­engraftment that peaks around day +7, gradually disappearing thereafter.

Later, based on the observation that IL-2 systemic administration favors T regulatory cells (Treg) expansion, Bachanova et al. [26] hypothesizing that the *in vivo* depletion of Tregs would facilitate adoptively transferred NK cells persistence and expansion, IL-2 conjugated with diphtheria toxin (IL-2 DT) was given one day before NK cell infusion. That strategy succeeded in obtaining higher peripheral blood NK cell persistence and expansion along with clinical enhanced efficacy overall response rate (ORR) of 53% as compared to their previous trial with IL-2 alone. They also showed that IL-15 serum level was increased after lymphodepletion and correlated with successful donor NK-cell expansion. Next, the same group, this time utilizing overnight incubation with IL-15, published their experience testing post-infusion IL-15 for the treatment of R/R AML [27]. IL-15 was administered either intravenously (IV) or subcutaneously (SC) to two consecutive cohorts. In the IV cohort, the primary end point was IL-15 maximum tolerate dose (MTD). In a subsequent cohort in the same study, SC IL-15 was administered following the NK-cell infusion. On the 7th day after infusion, 100% of circulating NK cells were from donor origin, and at day 14 donor NK cells were 42% and 33% of total cells in the IV and SC cohorts, respectively. In vivo NK-cell expansion was documented in 35% of the IV group of patients and in 27% of the SC cohort of patients. ORR was 35% (no difference between IV and SC), and 11 of the 40 evaluable patients went on to HSCT. However, in the SC cohort, there was one severe adverse effect (SAE) grade 5 caused by cytokine release syndrome (CRS) and neurotoxicity, and 56% of the patients had documented CRS while no CRS was observed in the IV cohort. Although the authors succeed in obtaining engraftment, expansion, and better persistence, there was a lack of correlation between *in vivo* NK cell persistence, expansion, and/or toxicity and clinical activity.

Curti et al. [28] reported the results of NK-cell immunotherapy in elderly high-risk AML patients. In this study, patients received one infusion of a non-activated, cryopreserved NK cell product followed by subcutaneous administration of IL-2 thrice weekly for six doses. Donor NK cells in PB and BM peaked at day 10 and 5, respectively, and PB donor chimerism varied from 2% to 18% in the evaluable patients. Complete remission (CR) was observed in one of the five R/R AML patients, while two patients in molecular relapse achieved molecular CR. Among the six patients treated in CR, three were alive and disease free 34, 32, and 18 months after NK cell therapy.

Increasing the NK cell product activity and better *in vitro* expansion has been pursued for many years. Harada et al. [29] published a preclinical study testing cells from a Wilms tumor cell line (WFWT) as feeders for *in vitro* activation of NK cells. After the end of the culture, more than 70% of the NK cell population was CD56^+^CD16^-^ and expressed additional phenotypic markers of an immature NK cell. However, the expanded NK cells killed not only fresh WFWT cells but also MHC class I-expressing autologous brain tumor cells. In a clinical study utilizing the same cell expansion platform, IV infused cells lead to a remarkable glioblastoma regression in two patients [29]. Later, Fujisaki et al. developed a technical platform utilizing irradiated K562 cells genetically modified to express membrane bound IL-15 and 4-1BB (mbIL-15K562) [30], and Denman et al., genetically modified K562 cells to express membrane bound IL-21 instead (mbIL-21 K562) [16], both obtaining a clinical grade expansion of highly active cytotoxic NK cells.

Vela et al. [31] utilized the mbIL-15K562 platform to *in vitro* expand haploidentical NK cells in a heavily pretreated population of pediatric patients with refractory T-ALL, AML, B-ALL, and biphenotypic AL. Reinduction regimens, the majority myeloablative, were administered twice. In cohort 1, one NK cells infusion (day 0), and in cohort 2 two NK cells infusions at day 0 and day 7 of each cycle, were administered. ORR, CR^MRD-^, and CR^MRD+^ (MRD for minimal residual disease) were 78%, 39%, and 39%, respectively. Donor NK cell engraftment was observed in 46.7% of the patients, and NK cell peaked at day 7. However, PB lymphocyte reconstitution or engraftment did not correlate with clinical response – 3 of the eight patients without donor chimerism achieved MRD negativity. There was no difference between the two cohorts. Remarkably, 62.5% of the patients who went on to HSCT after the end of the trial had received high-intensity chemotherapy, while none submitted to Flu/Cy were able to achieve it.

Utilizing mbIL21 K562 cells, we were able to consistently grow a pure (90%) population of CD56^bright^CD16^bright^ NK cells, at clinical grade numbers, enough to infuse a median of 2.85 × 10^9^ [IQ, 2.54–2.94] of highly activated CD56^bright^CD16^bright^ NK cells/patient [32]. These ‘artificial’ dual function Double Bright NK cells (DBNK) obtained in our culture condition express an array of tissue homing ligands, such as CXCR3 for liver or inflammation, CXCR4 for bone marrow, and ITGA4 and NCAM1 for central nervous system. We investigated the infusion of haploidentical DBNK cells to treat a very high-risk cohort of 13 R/R AML patients with a median ECOG of 3, relapsed/refractory to a median of five lines of treatment including HSCT in 69%. The ORR was 78.6%, of which 50% were CR. Patients received FLAG chemotherapy followed by six infusions of DBNK cells ranging from 10^6^ to 10^7^/kg/infusion without exogenous interleukin. The rationale for the treatment regimen was that repeated delivery of large NK cells doses would ensure cell activity across a 14-day period, avoiding exogenous interleukin side effects. Interestingly, we were not only able to document CNS responses with CR or PR in all four patients with CNS disease (including one with a probable fungal brain infection) but also an increasing number of NK cells that, despite the several infusions up to day 14, peaked at day +7, outnumbered by T cells in most responding patients [32]. On the other hand, there was a predominant NK cell peripheral blood expansion in three patients. One with steroid-responsive severe anemia probably related to DBNK cell immunotherapy; one refractory to two haploidentical SCT with different donors with an impressive peripheral blood NK cell expansion that turned out to be active in promoting DC activation of Th17 lymphocytes [33], without anti-leukemia activity. The third patient treated on a compassionate basis because of a severe resistant *Escherichia coli* ascending cholangitis did not respond and died of progressive AML several weeks later with significant clinical improvement of his cholangitis. No cytokine storm was observed, and all adverse events were manageable with no related death. As others have shown, we did not observed correlation between NK cell peripheral blood number or persistence and clinical efficacy. It is important to point out that we did not test for donor chimerism, so are unable to be sure if the striking in vivo NK cell expansion observed in some patients was of autologous or donor origin.

Utilizing cord blood derived *in vitro* activated NK cells, Dolstra et al. treated 11 patients with high-risk AML in CR obtaining a favorable disease-free survival (DFS) that remains to be confirmed in an awaited follow up [34]. Björklund et al. treated 16 R/R AML patients and obtained 25% CR, and a third of the patients were able to go on to HSCT [35].

It must be pointed out that in most of the revised studies (Table 1) although NK cells were selected for best donor according to KIR mismatch or the presence of KIR B haplotype, NK-cell alloreactivity did not influence clinical efficacy. This lack of correlation could be attributed to the relatively small population of patients treated in each study or the degree of *in vitro* NK cells activation. For R/R AML, as can be seen in Table 1, the transient clinical improvement should be utilized as a bridge to HSCT.

### GVL effect without SCT

Outside the R/R AML setting, Nguyen et al. published the results of a phase II study, including 21 intermediate-risk AML patients after completion of 4—5 cycles of chemotherapy. All patients were in CR at enrollment and received 1 infusion of fresh KIR mismatched haploidentical NK cells. A low- to intermediate-intensity lymphodepletion was started at day 7, followed by IL-2 1 million IU SC beginning on day 1 for a total of six doses. The primary endpoint was to compare event-free survival to a cohort of 55 patients who completed chemotherapy, were in first CR, but did not receive NK cells. Of the 21 patients who received NK cells, a transient engraftment of donor cells (median donor chimerism of 4%) was observed in 19 patients. When compared to the control group, the adoptive transfer of NK cells did not improve EFS or OS rates [36]. The hypothesis that HSCT could be replaced by adoptively transferred NK cells without GVHD was not corroborated in their study design.

## Discussion

In R/R AML, the adoptive transfer of NK cells without HSCT, NK cell transient engraftment and persistence has been repeatedly shown to be independent of the lymphodepletion intensity, cellular product activation, the number of cells infused, or the utilization of interleukin post infusion (Table 1). In this setting, better clinical activity, albeit without NK cell persistence, was observed in those trials that utilized in vitro activated cellular products [31, 32, 37]. On the other hand, in the trials that utilized genetically modified K562 cells as feeders for *in vitro* activation and expansion, the high-intensity lymphodepletion that was utilized made it impossible to know if *in vitro* activated NK cells or lymphodepletion or both, influenced results. As pointed out above, the disappointing results reported by the St. Jude group [36] could be attributed to the lack of NK cell *in vitr*o activation and/or the low-intensity lymphodepletion utilized.

Lymphodepletion intensity is still an unsettled matter. NK cells’ early recovery after HSCT was recently confirmed in a very interesting biosystem model study utilizing machine learning to examine transcriptome changes in lymphocyte subsets that predict major transplant outcomes [38]. This study showed that a favorable cytokine profile leads to a higher overall survival (OS) rate and progression free survival (PFS). The known early NK-cell recovery after HSCT could be related to what had been suggested by Steven Rosenberg’s group when describing the need for lymphodepletion before the adoptive transfer of T lymphocytes to treat melanoma [39]. According to them, myeloablative regimens or the almost absence of normal cells could induce a favorable milieux for lymphocyte expansion. In agreement with both above-mentioned studies, Hirayama et al. [40] recently showed that a favorable cytokine profile induced by lymphodepletion is associated with better clinical outcomes and tend to be obtained by higher dose lymphodepletion. On the other hand, there is no apparent difference in clinical activity between different regimens of low to moderate intensity [41].

The ideal number of NK cells infused or the number of infusions remains to be defined; in our study, the patient who presented the highest NK cell peripheral blood expansion with no anti-leukemia effect received only three of the six planned infusions, suggesting that the total number of cells infused as well as the number of infusions might be less important for *in vivo* expansion than recipient-related factors. Another unsettled matter is the *in vivo* persistence and expansion of adoptively transferred NK cells. As can be seen in Table 1, and as mentioned above, transient engraftment and persistence of donor NK cells has been repeatedly shown to be independent of all explored strategies.

Host immune effectors could reject transferred NK cells and explain the short persistence, however, most studies utilized only one infusion, and its kinetics was equally similar in the few multiple infusions’ studies; likewise, unrelated, non-HLA matched, *off the shelf* NK cells were shown to have the same kinetics as the infused haploidentical NK cells, and antibodies against donor class I or II HLA antigens were not detected in any recipient [42]. Taken together, these results suggest that NK cell rejection is a less likely explanation for the transient peripheral blood persistence. Finally, T cells expansion in the favorable cytokine milieux obtained by lymphodepletion could explain host T cells rejection of donor NK cells, however, as mentioned above, better cytokine profile is related to better clinical outcomes, suggesting that T cells immune recovery goes along with a better anti-tumor activity.

Based on these findings, we could hypothesize that, being an innate immune effector cell designed to alert the adaptive immune system through the secretion of cytokines, chemokines, and growth factors, NK cell kinetics in these trials were very much in accordance with their original description. After around 7 days of NK cell burst, adaptive immune effectors surpass NK cell numbers in an effective immune response [1]. We also could question if non-genetically modified NK cells should behave as CAR-T cells, for which peripheral blood expansion is a hallmark and is related to anti-tumor activity and severe adverse events. T lymphocytes are designed to be immune effectors that upon clonal expansion establish a robust memory and antibody production. Non-genetically modified NK-cell immunotherapy should aim to get the best T-cell response possible.

In conclusion, clinical trials studying non-genetically modified NK cell anti-leukemia activity have repeatedly showed that this activity is independent of peripheral blood NK cell persistence and expansion. It is possible that attempts to augment non-genetically modified NK cell peripheral blood expansion and persistence could jeopardize an appropriate and effective stimulation of the anti-tumor adaptive immune response at its own microenvironment, the primary role of innate effectors.

**Figure 1.**
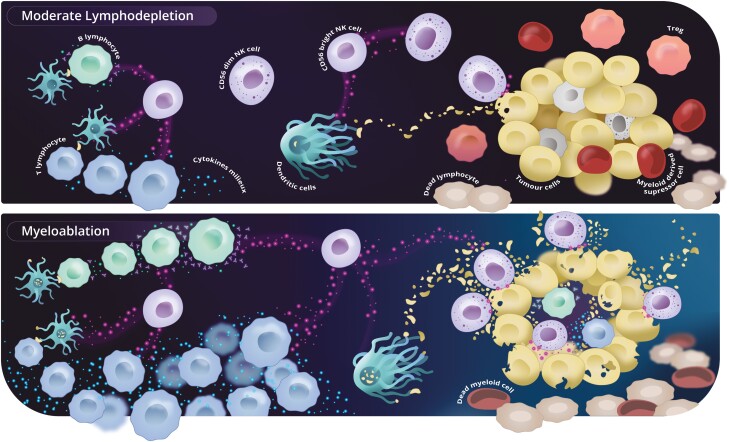
Possible mechanisms of non-genetically modified NK cells transferred activity. In the upper panel, a moderate lymphodepletions is accompanied by a less favorable microenvironment milieux and some reminiscent lymphocytes and myeloid derived suppressor cells that protect malignant cells from being significantly destroyed by the transferred lymphocytes. In the lower panel, a myeloablative lymphodepletion gives rise to a favorable milieux and a more profound depletion of suppressor cells facilitating transferred NK cells local activation and tumor cell destruction that derived molecules are captured by dendritic cells and presented to the adaptive immune system. CD56bright or regulatory NK cells, also activated in such a milieux, stimulate innate and adaptive immune system directly, cell by cell, or through its cross talking with DC cells, resulting in a robust adaptive immune response. An eventual tumor infiltrating lymphocyte can be activated in such a favorable environment. Professional illustration by Clara Heinrich.

## Data Availability

The author can confirm that all relevant data are included in the article and/or its references.

## Abbreviations

AL: Acute Leukemia
AML: Acute Myeloid Leukemia
B-ALL: B cell Acute Lymphocytic Leukemia
BM: Bone Marrow
CNS: Central Nervous System
CR: complete remission
CR^MDR-^: Complete Remission with no measurable Minimal Residual Disease
CR^MDR+^: Complete Remission with measurable Minimal Residual Disease
CRS: Cytokine Release Syndrome
DBNK: Double Bright NK cell
DC: Dendritic Cells
DFS: Disease Free Survival
E. Coli: Escherichia Coli
ECOG: Eastern Cooperative Oncology Group performance status scale
EFS: Event Free Survival
FLAG: Fludarabine + Arabinoside C + Granulocyte Colony-stimulating Factor
Flu/Cy: Fludarabine and Cyclophosphamide
GSF: Granulocyte Colony-stimulating Factor
GVHD: Graft Versus Host Disease
GVL: Graft versus Leukemia
HHSCT: Haploidentical Stem Cell Transplantation
HLA: human leucocyte antigen
HSCT: Hematopoietic Stem Cell Transplantation
IFN-?: Interferon gamma
IL-15: Interleukin 15
IL-2 DT: Interleukin 2 conjugated with Diphtheria Toxin
IL-2: Interleukin 2
IV: Intravenous
KIR: Killer Immunoglobulin Like Receptors
KIR: MM Killer Immunoglobulin like Receptors Mismatch
mbIL-15K562: membrane bound IL-15 K562 cell line
mbIL-21K562: membrane bound IL-15 K562 cell line
MDR: measurable Minimal Residual Disease
MHC: Major Histocompatibility Complex
MTD: Maxime Tolerate Dose
NK: natural killer
ORR: Overall Response Rate
OS: Overall survival
PB: Peripheral Blood
PR: Partial Remission
R/R AML: Relapse or Refractory Acute Myeloid Leukemia
SAE: Severe Adverse Event
SC: subcutaneous
SCT: Stem cell Transplantation
T-ALL: T cell Acute Lymphocytic Leukemia
TGF-β: Transforming Growth Factor beta
Treg: regulatory T cell
trNK: Tissue Resident NK cell
uNK: uterine NK cells
WFWT: Wilms Tumor cell line

## Animal research adheres to the ARRIVE guidelines

This study does not involve animal research.

## Permission to reproduce

The author states that the reproduction of short extracts of text were used for the purpose of quotation and/or evidenced scholarly criticism or review and exist in the public domain. The author conclusions are free to be reproduced.

## References

[1] Ferlazzo G , Münz C. NK cell compartments and their activation by dendritic cells. J Immunol 2004; 172(3):1333–9. 10.4049/jimmunol.172.3.1333 14734707

[2] Adams NM , Diaz-Salazar C, Dang C et al. Cutting edge: heterogeneity in cell age contributes to functional diversity of NK cells. J Immunol 2021; 206(3):465–70. 10.4049/jimmunol.2001163 33443057 PMC8915861

[3] Vivier E , Raulet DH, Moretta A et al. Innate or adaptive immunity? The example of natural killer cells. Science 2011; 331(6013):44–9. 10.1126/science.1198687 21212348 PMC3089969

[4] Dogra P , Rancan C, Ma W et al. Tissue determinants of human NK cell development, function, and residence. Cell 2020; 180(4):749–763.e13. 10.1016/j.cell.2020.01.022 32059780 PMC7194029

[5] Ljunggren HG , Kärre K. In search of the ‘missing self’: MHC molecules and NK cell recognition. Immunol Today 1990; 11(7):237–44. 10.1016/0167-5699(90)90097-s 2201309

[6] Chretien AS , Devillier R, Granjeaud S et al. High-dimensional mass cytometry analysis of NK cell alterations in AML identifies a subgroup with adverse clinical outcome. Proc Natl Acad Sci USA 2021; 118(22):e2020459118 34050021 10.1073/pnas.2020459118 PMC8179170

[7] Wang H , Tao Q, Wang Z et al. Circulating monocytic myeloid-derived suppressor cells are elevated and associated with poor prognosis in acute myeloid leukemia. J Immunol Res 2020; 2020:7363084. 10.1155/2020/7363084 33415170 PMC7769680

[8] Campbell JJ , Qin S, Unutmaz D et al. Unique subpopulations of CD56+ NK and NK-T peripheral blood lymphocytes identified by chemokine receptor expression repertoire. J Immunol 2001; 166(11):6477–82. 10.4049/jimmunol.166.11.6477 11359797

[9] Scharenberg M , Vangeti S, Kekäläinen E et al. Influenza A virus infection induces hyperresponsiveness in human lung tissue-resident and peripheral blood NK cells. Front Immunol 2019; 10:1116. 10.3389/fimmu.2019.01116 31156653 PMC6534051

[10] Jinushi M , Takehara T, Tatsumi T et al. Natural killer cell and hepatic cell interaction via NKG2A leads to dendritic cell-mediated induction of CD4 CD25 T cells with PD-1-dependent regulatory activities. Immunology 2007; 120(1):73–82. 10.1111/j.1365-2567.2006.02479.x 17052247 PMC2265878

[11] Morandi B , Bougras G, Muller WA et al. NK cells of human secondary lymphoid tissues enhance T cell polarization via IFN-gamma secretion. Eur J Immunol 2006; 36(9):2394–400. 10.1002/eji.200636290 16917961

[12] Law BMP , Wilkinson R, Wang X et al. Interferon-γ production by tubulointerstitial human CD56. Kidney Int 2017; 92(1):79–88. 10.1016/j.kint.2017.02.006 28396119

[13] Gaynor LM , Colucci F. Uterine natural killer cells: functional distinctions and influence on pregnancy in humans and mice. Front Immunol 2017; 8:467. 10.3389/fimmu.2017.00467 28484462 PMC5402472

[14] Chidrawar SM , Khan N, Chan YL et al. Ageing is associated with a decline in peripheral blood CD56bright NK cells. Immun Ageing 2006; 3:10. 10.1186/1742-4933-3-10 17134511 PMC1702551

[15] Varchetta S , Mele D, Oliviero B et al. Unique immunological profile in patients with COVID-19. Cell Mol Immunol 2021; 18(3):604–12. 10.1038/s41423-020-00557-9 33060840 PMC7557230

[16] Denman CJ , Senyukov VV, Somanchi SS et al. Membrane-bound IL-21 promotes sustained ex vivo proliferation of human natural killer cells. PLoS One 2012; 7(1):e30264. 10.1371/journal.pone.0030264 22279576 PMC3261192

[17] Horowitz MM , Gale RP, Sondel PM et al. Graft-versus-leukemia reactions after bone marrow transplantation. Blood 1990; 75(3):555–62. 2297567

[18] Ruggeri L , Capanni M, Urbani E et al. Effectiveness of donor natural killer cell alloreactivity in mismatched hematopoietic transplants. Science 2002; 295(5562):2097–100. 10.1126/science.1068440 11896281

[19] Dickinson AM , Norden J, Li S et al. Graft-versus-leukemia effect following hematopoietic stem cell transplantation for leukemia. Review Front Immunol 2017; 8:496. 10.3389/fimmu.2017.00496 28638379 PMC5461268

[20] Hattori N , Saito B, Sasaki Y et al. Status of natural killer cell recovery in day 21 bone marrow after allogeneic hematopoietic stem cell transplantation predicts clinical outcome. Biol Blood Marrow Transplant 2018; 24(9):1841–7. 10.1016/j.bbmt.2018.05.007 29753837

[21] Ciurea SO , Al Malki MM, Kongtim P et al. The European Society for Blood and Marrow Transplantation (EBMT) consensus recommendations for donor selection in haploidentical hematopoietic cell transplantation. Bone Marrow Transplant 2020; 55(1):12–24. 10.1038/s41409-019-0499-z 30833742

[22] Venstrom JM , Pittari G, Gooley TA et al. HLA-C-dependent prevention of leukemia relapse by donor activating KIR2DS1. N Engl J Med 2012; 367(9):805–16. 10.1056/NEJMoa1200503 22931314 PMC3767478

[23] Boudreau JE , Giglio F, Gooley TA et al. KIR3DL1/HLA-B subtypes govern acute myelogenous leukemia relapse after hematopoietic cell transplantation. J Clin Oncol 2017; 35(20):2268–78. 10.1200/JCO.2016.70.7059 28520526 PMC5501362

[24] Schetelig J , Baldauf H, Heidenreich F et al. External validation of models for KIR2DS1/KIR3DL1-informed selection of hematopoietic cell donors fails. Blood 2020; 135(16):1386–95. 10.1182/blood.2019002887 31932846 PMC7162689

[25] Miller JS , Soignier Y, Panoskaltsis-Mortari A et al. Successful adoptive transfer and in vivo expansion of human haploidentical NK cells in patients with cancer. Blood 2005; 105(8):3051–7. 10.1182/blood-2004-07-2974 15632206

[26] Bachanova V , Cooley S, Defor TE et al. Clearance of acute myeloid leukemia by haploidentical natural killer cells is improved using IL-2 diphtheria toxin fusion protein. Blood 2014; 123(25):3855–63. 10.1182/blood-2013-10-532531 24719405 PMC4064329

[27] Cooley S , He F, Bachanova V et al. First-in-human trial of rhIL-15 and haploidentical natural killer cell therapy for advanced acute myeloid leukemia. Blood Adv 2019; 3(13):1970–80. 10.1182/bloodadvances.2018028332 31266741 PMC6616260

[28] Curti A , Ruggeri L, D’Addio A et al. Successful transfer of alloreactive haploidentical KIR ligand-mismatched natural killer cells after infusion in elderly high risk acute myeloid leukemia patients. Blood 2011; 118(12):3273–9. 10.1182/blood-2011-01-329508 21791425

[29] Harada H , Saijo K, Watanabe S et al. Selective expansion of human natural killer cells from peripheral blood mononuclear cells by the cell line, HFWT. Jpn J Cancer Res 2002; 93(3):313–9. 10.1111/j.1349-7006.2002.tb02174.x 11927014 PMC5926967

[30] Fujisaki H , Kakuda H, Shimasaki N et al. Expansion of highly cytotoxic human natural killer cells for cancer cell therapy. Cancer Res 2009; 69(9):4010–7. 10.1158/0008-5472.CAN-08-3712 19383914 PMC2716664

[31] Vela M , Corral D, Carrasco P et al. Haploidentical IL-15/41BBL activated and expanded natural killer cell infusion therapy after salvage chemotherapy in children with relapsed and refractory leukemia. Cancer Lett 2018; 422:107–17. 10.1016/j.canlet.2018.02.033 29477379

[32] Silla L , Valim V, Pezzi A et al. Adoptive immunotherapy with double-bright (CD56 bright/CD16 bright) expanded natural killer cells in patients with relapsed or refractory acute myeloid leukaemia: a proof-of-concept study. Br J Haematol 2021; 84(710):1–12. 10.1111/bjh.17751 34490616

[33] Clavijo-Salomon MA , Salcedo R, Roy S et al. Human NK cells prime inflammatory DC precursors to induce Tc17 differentiation. Blood Adv 2020; 4(16):3990–4006. 10.1182/bloodadvances.2020002084 32841340 PMC7448590

[34] Dolstra H , Roeven MWH, Spanholtz J et al. Successful transfer of umbilical cord blood CD34. Clin Cancer Res 2017; 23(15):4107–18. 10.1158/1078-0432.CCR-16-2981 28280089

[35] Björklund AT , Carlsten M, Sohlberg E et al. Complete remission with reduction of high-risk clones following haploidentical NK-cell therapy against MDS and AML. Clin Cancer Res 2018; 24(8):1834–44. 10.1158/1078-0432.CCR-17-3196 29444931

[36] Nguyen R , Wu H, Pounds S et al. A phase II clinical trial of adoptive transfer of haploidentical natural killer cells for consolidation therapy of pediatric acute myeloid leukemia. J ImmunoTher Cancer 2019; 7(1):8–1. 30894213 10.1186/s40425-019-0564-6 PMC6425674

[37] Romee R , Rosario M, Berrien-Elliott MM et al. Cytokine-induced memory-like natural killer cells exhibit enhanced responses against myeloid leukemia. Sci Transl Med 2016; 8(357):357–123. 10.1126/scitranslmed.aaf2341 PMC5436500 27655849

[38] McCurdy SR , Radojcic V, Tsai HL et al. Signatures of GVHD and relapse after posttransplant cyclophosphamide revealed by immune profiling and machine learning. Blood 2022; 139(4):608–23. 10.1182/blood.2021013054 34657151 PMC8796655

[39] Dudley ME , Yang JC, Sherry R et al. Adoptive cell therapy for patients with metastatic melanoma: evaluation of intensive myeloablative chemoradiation preparative regimens. J Clin Oncol 2008; 26(32):5233–9. 10.1200/JCO.2008.16.5449 18809613 PMC2652090

[40] Hirayama AV , Gauthier J, Hay KA et al. The response to lymphodepletion impacts PFS in patients with aggressive non-Hodgkin lymphoma treated with CD19 CAR T cells. Blood 2019; 133(17):1876–87. 10.1182/blood-2018-11-887067 30782611 PMC6484391

[41] Nissani A , Lev-Ari S, Meirson T et al. Comparison of non-myeloablative lymphodepleting preconditioning regimens in patients undergoing adoptive T cell therapy. J ImmunoTher Cancer 2021; 9(5):e001743452–548. 10.1136/jitc-2020-001743 PMC8127974 33990415

[42] Otegbeye F , Cooper B, Caimi P et al. A phase I study to determine the maximum tolerated dose of ex vivo expanded natural killer cells derived from unrelated, HLA-disparate adult donors. Transplant Cell Ther. 2022;28(5):250:1-8. 10.1016/j.jtct.2022.02.008 PMC9489303 35172204

