# Correction to: Peripheral blood persistence and expansion of transferred non-genetically modified Natural Killer cells might not be necessary for clinical activity

**DOI:** 10.1093/immadv/ltad003

**Published:** 2023-02-18

This is a correction to: Lucia Silla, Peripheral blood persistence and expansion of transferred non-genetically modified Natural Killer cells might not be necessary for clinical activity, *Immunotherapy Advances*, Volume 3, Issue 1, 2023, https://doi.org/10.1093/immadv/ltac024

In the originally published version of this manuscript, errors were introduced during production steps. **Introduction** (p2, column b), the subheading should read: “**NK cell anti-tumour activity**” instead of: “**NK zcell anti-tumor activity**”. In section **NK cell adoptive immunotherapy**, p3, column b, second sentence should read: “IL is usually administered during the 14 days following cells infusion, aiming to activate and expand the donor NK cells *in vivo*.” instead of: “Interleukin (IL) is usually administered during the 14 days following cells infusion, aiming to activate and expand the donor NK cells *in vivo*.” In section **GVL effect without SCT**, p6, column a, third sentence should read: “A low- to intermediate-intensity lymphodepletion was started at day -7, followed by IL-2 1 million IU SC beginning on day -1 for a total of six doses.” instead of: “A low- to intermediate-intensity lymphodepletion was started at day 7, followed by IL-2 1 million IU SC beginning on day 1 for a total of six doses.”

The publisher regrets these errors. They have been emended in the article.

